# Colchicine has Dose‐Dependent Therapeutic Effects in a LPS‐Induced Experimental Endotoxemia Model

**DOI:** 10.1002/prp2.70239

**Published:** 2026-04-06

**Authors:** Meliksah Demir, Ayse Yesim Gocmen, Esin Ozcelebi, Zuleyha Doganyigit, Aslı Okan Oflamaz, Alper Bektas Iskit

**Affiliations:** ^1^ Department of Medical Pharmacology Hacettepe University, Faculty of Medicine Ankara Turkey; ^2^ Department of Histology and Embryology Bozok University Yozgat Turkey

**Keywords:** anti‐inflammatory effect, colchicine, LPS‐induced endotoxemia, mesenteric artery blood flow, multiple organ damage

## Abstract

Colchicine is an ancient medication that has long been used for anti‐inflammatory properties. Because of unfavorable and toxic consequences of colchicine, selecting an appropriate dose is critical. The effects of three different doses (0.5, 1 or 5 mg/kg) of colchicine were evaluated. To stimulate systemic inflammation, we created an LPS‐induced experimental endotoxemia model with 1 mg/kg LPS intraperitoneal injection. Possible therapeutic effects of colchicine were investigated on cytokines, decreased mesenteric artery blood flow and histopathological damage scores. Treatment with 1 mg/kg colchicine reduced the pro‐inflammatory cytokines IL‐1β, TNF‐α, IL‐8, and IL‐17 to levels comparable to the control group and attenuated the damage caused by LPS in liver and spleen tissues. At a dose of 0.5 mg/kg, colchicine was not able to decrease cytokine levels to those of the control group. In contrast, administration of 5 mg/kg colchicine ameliorated mesenteric blood flow; however, this higher dose caused an increase in cytokine levels. Among three different colchicine doses, 1 mg/kg intraperitoneal dose significantly improved the inflammatory indices and may be considered a suitable option for anti‐inflammatory treatment.

## 1 INTRODUCTION

Colchicine is extracted from the seeds of the *Colchicum autumnale* plant and has been used for over 3000 years to relieve joint pain, as mentioned in the Ebers Papyrus, an ancient Egyptian medical papyrus. Today, the FDA has approved colchicine as a treatment for gout flares and FMF (Familial Mediterranean Fever). Colchicine is also used off‐label to treat liver cirrhosis, primary biliary cirrhosis, Behçet's disease, and pericarditis. Colchicine's cardiovascular effects are also being investigated. Colchicine, which binds to tubulin and inhibits microtubule polymerization in cells, reduces inflammation and cell division during metaphase [1]. Colchicine exerts anti‐inflammatory effects by inhibiting the NLRP3 inflammasome and reducing cytokine production [2]. Colchicine has a narrow therapeutic window, which limits its clinical applicability. Although colchicine poisoning is uncommon, it can be fatal. Therefore, maintaining a consistent dosing range and plasma concentration throughout treatment is essential.

In experimental research on systemic inflammation, intraperitoneal administration of lipopolysaccharide (LPS) is widely used to induce endotoxemia, reproducing key features of early sepsis such as a surge in pro‐inflammatory cytokines, endothelial dysfunction, microcirculatory failure and organ injury. This LPS model induces non‐infectious endotoxemia and reproduces several inflammatory and hemodynamic features observed in septic shock, but it is not a true infectious sepsis model. In endotoxemic murine models, LPS challenge has been shown to reduce mesenteric blood flow, increase vascular resistance, impair vascular reactivity to vasoconstrictors, and cause structural and functional damage in organs such as the liver and spleen, effects that are critically modulated by nitric oxide and endothelin pathways [3, 4, 5, 6]. Colchicine, a microtubule‐disrupting drug with established anti‐inflammatory and anti‐neutrophil effects, has recently been reported to attenuate sepsis‐induced liver injury and to ameliorate LPS‐induced lung injury by reducing pro‐inflammatory cytokine levels and inhibiting MAPK signaling [7, 8]. However, in an LPS‐induced endotoxemia model, the dose dependent effects of colchicine on the systemic cytokine response and concurrent hepatic and splenic tissue injury have not been fully elucidated. Therefore, taking previous studies into consideration, three different doses of colchicine (0.5 mg/kg, 1 mg/kg, and 5 mg/kg) were selected [9, 10, 11, 12]. The anti‐inflammatory effect of colchicine was evaluated using interleukin levels measured in serum and in tissue samples from the liver, spleen and heart. In addition, blood flow in the mesenteric artery was examined to assess its effect on perfusion changes. Furthermore, histological damage scores of the liver and spleen were assessed to determine the protective effect of colchicine.

## 2 Materials and Methods

The protocols were carried out at the laboratories of the Department of Medical Pharmacology at Hacettepe University Faculty of Medicine, with consent from the Hacettepe University Animal Ethics Committee (Approval No: 2021/06–20). Male Wistar Albino rats weighing 250–300 g and aged 8–10 weeks were utilized. The animals were housed in a temperature‐controlled facility with a 12‐h dark/12‐h light cycle at 21°C ± 2°C. Before beginning the experimental process, the animals were exposed to the environment for 15 days to allow for acclimatization. They were fed a regular rodent laboratory diet and allowed unlimited access to water. This work followed the National Research Council's Guiding Principles in the Care and Use of Laboratory Animals, as well as the ARRIVE Guidelines.

### 2.1 Experimental Protocols

Animals were fasted for 16 h before the procedure. LPS (O55:B5) at a dose of 1 mg/kg and colchicine at doses of 0.5, 1, and 5 mg/kg were dissolved in saline solution [9, 11, 12, 13]. LPS (1 mg/kg, i.p.) was injected 4 h before sample collection, and colchicine (0.5, 1, or 5 mg/kg, i.p.) was administered 1 h after LPS. All parameters were evaluated 4 h after LPS injection (3 h after colchicine treatment), corresponding to the early phase of the cytokine response [14].

Eight experimental groups were established as follows: (1) Saline (ip) (Control), (2) LPS (1 mg/kg, ip) (LPS), (3) Saline + colchicine 0.5 mg/kg (0.5 mg/kg Col), (4) Saline + colchicine 1 mg/kg (1 mg/kg Col), (5) Saline + colchicine 5 mg/kg (5 mg/kg Col), (6) LPS + colchicine 0.5 mg/kg (LPS + 0.5 mg/kg Col), (7) LPS + colchicine 1 mg/kg (LPS + 1 mg/kg Col), and (8) LPS + colchicine 5 mg/kg (LPS + 5 mg/kg Col). Each group initially consisted of six rats. However, in the LPS + colchicine 5 mg/kg group, two animals died during the experiment due to apparent cardiac toxicity and were therefore excluded from further analysis, resulting in a final sample size of *n* = 4 for this group and *n* = 6 for all other groups.

Sample size was determined using the resource equation method [15], taking into account the expected variability in biochemical, histopathological, and in vivo mesenteric blood flow measurements, as well as potential attrition due to model‐ and dose‐related mortality. Accordingly, a group size of six animals was considered appropriate to ensure reliable statistical evaluation. The number of animals used in this study is also consistent with previous reports employing similar LPS‐induced endotoxemia designs and hemodynamic and cytokine endpoints [16].

All rats were anesthetized with Chloral hydrate (400 mg/kg, ip) 4 h (240th minute) after LPS administration to measure mesenteric artery blood flow.

### 2.2 Measurement of Mesenteric Arterial Blood Flow Velocity

The surgical procedure applied in this study was based on a previously described method. Mesenteric arterial blood flow was measured using a Transonic T106 flowmeter with a 1RB probe (Transonic Systems Inc., USA), as reported by Ozer et al. [17]. Following midline laparotomy under anesthesia, the mesenteric artery was gently isolated, and the flow probe was positioned without disrupting perfusion [17]. The depth of anesthesia was evaluated by the response to a finger or skin pinch before initiating the surgical procedure. During the procedure, body temperature was monitored with a rectal thermometer probe and maintained at 37°C.

### 2.3 Collection and Storage of Samples

After measuring the mesenteric artery blood flow rate, the thorax was opened and blood was drawn by intracardiac puncture. After being centrifuged at 3000 rpm for 10 min, the serum separated from other blood materials was transferred to Eppendorf tubes and stored in a −80°C freezer.

Tissue samples taken from heart, liver, and spleen were placed in a 10% formaldehyde solution for histopathological damage score assessment after measurement of wet tissue weight. Collected tissues were taken to the Department of Histology and Embryology at Bozok University Faculty of Medicine for analysis. The remaining heart, liver, and spleen samples were placed in Eppendorf tubes and kept in a −80°C freezer.

### 2.4 Histopathological Examination of Tissue Sections

The spleen and liver samples obtained from control and experimental groups were fixed in 10% neutral buffered formalin, dehydrated through graded ethanol, cleared in xylene, and embedded in paraffin. Sections of 4 μm thickness were cut from the paraffin blocks using a sliding microtome (Leica SM2000R) and mounted on glass slides. The sections were stained with Harris hematoxylin and eosin (H&E) for routine histopathological evaluation and examined under a light microscope (Olympus BX53). To assess the degree of fibrosis, additional sections were stained with Mallory's trichrome and examined under a bright‐field microscope (Leica DM6000 B). Images were captured using a Leica DCF7000 T digital camera and recorded for analysis.

In liver sections, hepatocellular structural alterations, sinusoidal and central vein dilatation, fibrosis, vascular stasis, and inflammatory cell infiltration were evaluated. In spleen sections, sinusoidal dilatation, hemorrhage, inflammatory cell infiltration, and the presence of vacuolated and/or pyknotic damaged cells were assessed. Histopathological changes in both tissues were semi‐quantitatively scored as follows: 0 = absent (normal histological appearance), 1 = mild, 2 = moderate, and 3 = severe changes. For each animal, damage scores for each parameter were calculated based on the evaluation of 10 randomly selected microscopic fields per section. Histological evaluation was performed by a pathologist blinded to the experimental groups.

### 2.5 Measurement of Cytokine Levels in by Quantitative Enzyme Linked Immunosorbent Assay (ELISA)

Then they were divided into small pieces and washed several times with physiological serum, then homogenized in cold phosphate buffer (50 mM, pH 7.4) using a sonicator. The homogenate was centrifuged at 10000 x g for 15 min at 4° *C. ELISA* technique is used to determine cytokine (TNF‐α, IL‐1β, IL‐6, IL‐8, IL‐10 and IL‐17) concentrations according to the manufacturer's instructions.

### 2.6 Drugs

Lipopolysaccharide (LPS) belonging to the *Escherichia coli* O55:B5 serotype (L2880, Sigma‐Aldrich, St. Louis MO, USA), Colchicine (Sigma‐Aldrich, MO, USA), and Chloral hydrate (MERCK KGaA, Darmstadt, Germany) were used in the experiments.

### 2.7 Statistical Analysis

Data were demonstrated in mean ± standard error of the mean (SEM) and analyzed with GraphPad Prism 9. Statistical tests used for analysis and *n* values were expressed in the figure and table legends. Differences regarded as statistically significant when *p* value was < 0.05.

## 3 Results

### 3.1 Mesenteric Artery Blood Flow Measurement

**FIGURE 1:**
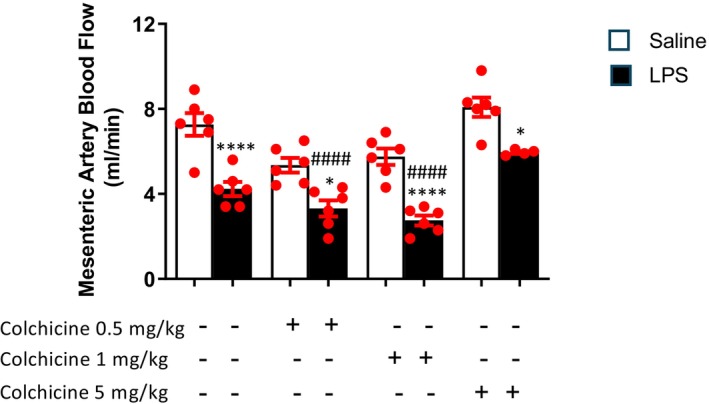
Changes in the mesenteric blood flow velocity dependent on colchicine treatment measured at the fourth hour after LPS injection. Data are presented as mean ± SEM. The intergroup comparison was performed using one‐way analysis of variance (ANOVA) followed by post hoc Tukey test (*n* = 6 animals per group, except LPS + 5 mg/kg colchicine group where *n* = 4, * indicates a significant difference with adjacent control groups, # indicates a significant difference compared to control group, Δ indicates a significant difference compared to LPS group (*, ^#^, ^Δ^ *p* < 0.05; **, ^##^, ^Δ Δ^ *p* < 0.01; ***, ^###^, ^Δ Δ Δ^ *p* < 0.001; ****, ^####^, ^Δ Δ Δ Δ^ *p* < 0.0001)).

In the LPS group, mesenteric arterial blood flow velocity decreased compared to the control group (Figure 1). Intraperitoneal colchicine injections 0.5 mg/kg and 1 mg/kg 1 h after endotoxin application did not ameliorate this decrease in mesenteric blood flow velocity compared to their adjacent control groups. There was no difference between the control group and the 5 mg/kg colchicine treatment group; however, colchicine treatment at a dose of 5 mg/kg resulted in a statistically significant difference with the adjacent LPS‐treated group.

### 3.2 Biochemical Markers

TNF‐α concentrations were given in Table 1. Compared to the control group, LPS administration significantly increased serum TNF‐α concentration. In the LPS‐induced experimental endotoxemia model, there was no statistical difference between the groups that received colchicine treatment at doses of 0.5, 1, or 5 mg/kg and the groups that received only 0.5, 1, or 5 mg/kg colchicine injection, respectively. At doses of 0.5 and 5 mg/kg, colchicine did not lower TNF‐α levels to the level of the control group and a statistically significant difference was detected between these doses of colchicine treatment groups and the control group. At the dose of 1 mg/kg, colchicine diminished the increase in TNF‐α level caused by LPS and reduced it to the levels of the control group. When the TNF‐α concentrations between treatment and control groups of the heart, spleen and liver tissue samples were compared, no statistically significant difference was found.

**TABLE 1:** Cytokine concentractions, measured at the fourth hour after LPS injection. Data are presented as mean ± SEM.

Group	TNF‐α	IL‐1β	IL‐8	IL‐17	IL‐6	IL‐10
Control (Saline)	396.63 ± 66.97	6.82 ± 0.63	153.13 ± 89.43	34.40 ± 4.56	107.14 ± 46.22	150.31 ± 22.22
LPS	704.56 ± 64.37****	43.41 ± 2.99****	613.48 ± 107.09***	56.09 ± 5.04****	275.19 ± 46.89****	256.51 ± 23.26***
0.5 mg/kg Col	453.26 ± 55.58	11.05 ± 1.46	170.97 ± 87.53	36.31 ± 4.03	94.85 ± 27.70	168.03 ± 23.47
LPS + 0.5 mg/kg Col	610.35 ± 45.99^##^	28.29 ± 0.94^####^	580.32 ± 84.38^##^	47.42 ± 2.56^#^	190.42 ± 24.51^###^	238.08 ± 17.90^##^
1 mg/kg Col	417.01 ± 22.86	12.05 ± 1.84	210.34 ± 56.98	35.91 ± 1.83	147.84 ± 25.31	161.15 ± 10.94
LPS + 1 mg/kg Col	453.70 ± 30.29	22.21 ± 0.87^####^	394.76 ± 47.52	41.52 ± 1.58	196.98 ± 10.61^###^	206.15 ± 17.86
5 mg/kg Col	623.93 ± 26.04	21.13 ± 1.32	325.00 ± 108.42	53.20 ± 4.76	226.82 ± 38.56^###^	198.13 ± 28.76
LPS + 5 mg/kg Col	727.60 ± 32.20^##^	34.52 ± 0.85^####^	664.32 ± 31.45^#^	55.15 ± 2.04^##^	278.27 ± 22.65^####^	270.27 ± 8.76^##^

*Note:* Group comparisons were made using one‐way ANOVA followed by post hoc Tukey test *n* = 6 animals per group, except LPS + 5 mg/kg colchicine group where *n* = 4, * represents comparison with difference from adjacent column, # represents comparison with control group, Δ represents comparison with LPS group (*, ^#^, ^Δ^ *p* < 0.05; **, ^##^, ^Δ Δ^ *p* < 0.01; ***, ^###^, ^Δ Δ Δ^ *p* < 0.001; ****, ^####^, ^Δ Δ Δ Δ^ *p* < 0.0001).

IL‐1β concentration measurements were shown in Table 1. In the LPS group, there was a statistically significant increase in serum IL‐1β concentration compared to the control group (86.82 ± 5.97 and 13.63 ± 1.26). All of the colchicine treatments decreased the level of IL‐1β compared to the LPS group, but only treatment with 1 mg/kg reduced it to the level of the control group. Although 5 mg/kg Col treatment decreased IL‐1β concentration compared to the LPS group, it had a statistically significant difference with only 5 mg/kg Col injected group. IL‐1β concentration in samples from liver, spleen and heart tissues did not show any statistically significant difference among only the LPS injected, colchicine treated and control groups.

As shown in Table 1, LPS administration significantly increased serum IL‐6 concentration compared to control group (*p* < 0.0001). 0.5 mg/kg and 1 mg/kg Col treatments decreased IL‐6 concentration caused by LPS. All three doses of colchicine could not reduce IL‐6 level to the level of the control group. There was no difference in IL‐6 concentrations between 5 mg/kg ip Col + LPS and 5 mg/kg Col groups. However, only 5 mg/kg Col injection increased IL‐6 levels compared to control group. The IL‐6 concentration in samples from liver and spleen tissues did not show any statistically significant difference between colchicine‐treated and control groups.

As it was shown in Table 1, LPS administration statistically significantly increased serum IL‐8 levels compared to the control group. There was no statistically significant difference between LPS + 1 mg/kg Col and the control group. 1 mg/kg Col treatment prevented the increase in IL‐8 concentration caused by LPS. However, treatments with 0.5 mg/kg and 5 mg/kg Col did not prevent the increase in IL‐8 concentration. When the IL‐8 concentrations between treatment and control groups of samples from heart, spleen and liver tissue were compared, no statistically significant difference was observed.

LPS administration significantly increased serum IL‐10 concentration compared to the control group as it was shown in Table 1 (*p* < 0.001). There was no statistically significant difference between LPS + 1 mg/kg Col and control groups. The increase in IL‐10 concentration was diminished at this dose. However, 0.5 mg/kg and 5 mg/kg Col treatments did not lower the increment in IL‐10 concentration caused by LPS. There was no statistically significant difference between treatment and control groups observed when IL‐10 concentrations in the heart, spleen and liver tissue samples are compared.

As it was demonstrated in Table 1, LPS administration significantly increased serum IL‐17 concentration compared to the control group. In LPS‐induced experimental endotoxemia model, there was no statistically significant difference between treatment groups and the groups only injected with same dose of colchicine. Colchicine at doses of 0.5 mg/kg and 5 mg/kg did not prevent the increase in IL‐17 concentrations in animals with LPS‐induced experimental endotoxemia. With 1 mg/kg Colchicine treatment, the increase in IL‐17 levels caused by LPS was prevented. No statistically significant difference between treatment and control groups was found when IL‐17 concentrations of heart, spleen and liver tissue samples were evaluated.

### 3.3 Histopathological Assessments of Liver and Spleen

#### 3.3.1 Liver

**FIGURE 2:**
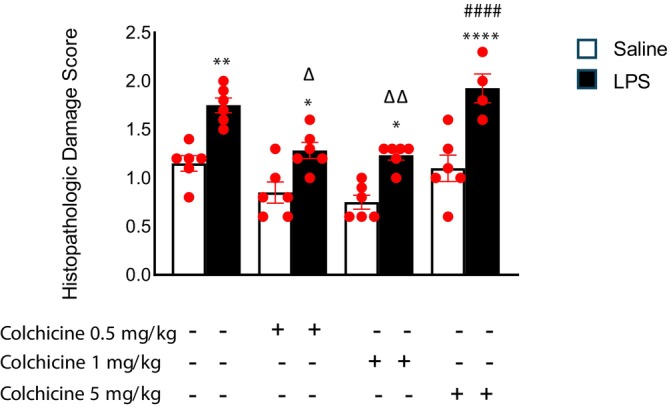
Change in histopathological damage score in the liver tissue after four hours of LPS injection, dependent on colchicine treatment. Data are presented as mean ± SEM. Group comparisons were made using one‐way ANOVA followed by post hoc Tukey test (*n* = 6 animals per group, except LPS + 5 mg/kg colchicine group where *n* = 4, * indicates significant difference from adjacent control groups, # indicates significant difference from control group, Δ indicates significant difference from LPS group. (*, ^#^, ^Δ^ *p* < 0.05; **, ^##^, ^Δ Δ^ *p* < 0.01; ***, ^###^, ^Δ Δ Δ^ *p* < 0.001; ****, ^####^, ^Δ Δ Δ Δ^ *p* < 0.0001)).

**FIGURE 3:**
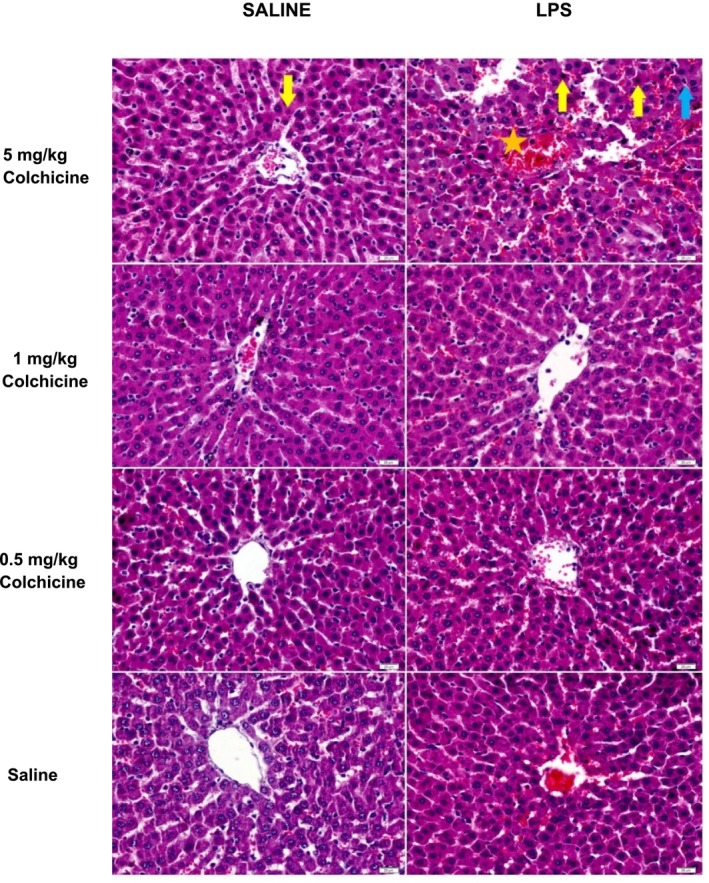
The effect of different colchicine doses on histopathological changes in a rat LPS induced endotoxemia model. Liver sections of treatment groups and healthy experimental animals were compared using the H&E staining method (yellow arrow: degenerated hepatocyte, orange star: bleeding, blue arrow: lymphocyte). Magnification 40×, bar = 20 μm.

The histopathological liver damage score was significantly increased in the LPS group compared with the control group (Figures 2 and 3). Treatment with colchicine at doses of 0.5 and 1 mg/kg significantly reduced the histopathological damage scores and restored them to levels comparable to those of the control group. In contrast, intraperitoneal administration of colchicine at 5 mg/kg did not attenuate the LPS‐induced liver damage (*p* < 0.001). According to the statistical analysis, no significant differences were observed in wet liver weights among the groups.

#### 3.3.2 Spleen

**FIGURE 4:**
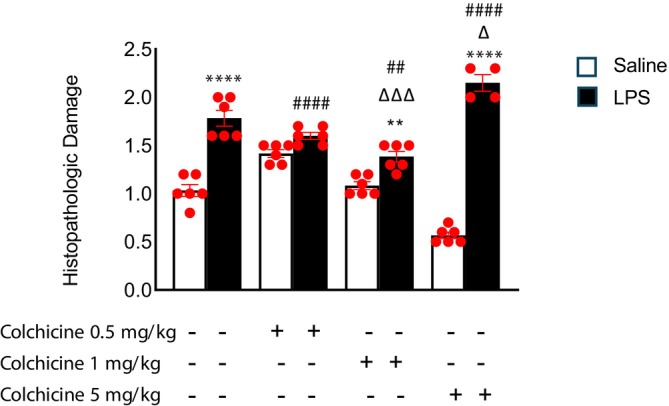
Histopathological damage score changes in spleen tissue due to colchicine treatment at the fourth hour after LPS injection. Data are presented as mean ± SEM. Group comparisons were made using one‐way ANOVA followed by post hoc Tukey test. (*n* = 6 animals per group, except LPS + 5 mg/kg colchicine group where *n* = 4, * indicates comparison with adjacent control groups, # indicates comparison with control group, Δ indicates comparison with LPS group. (*, ^#^, ^Δ^ *p* < 0.05; **, ^##^, ^Δ Δ^ *p* < 0.01; ***, ^###^, ^Δ Δ Δ^ *p* < 0.001; ****, ^####^, ^Δ Δ Δ Δ^ *p* < 0.0001)).

**FIGURE 5:**
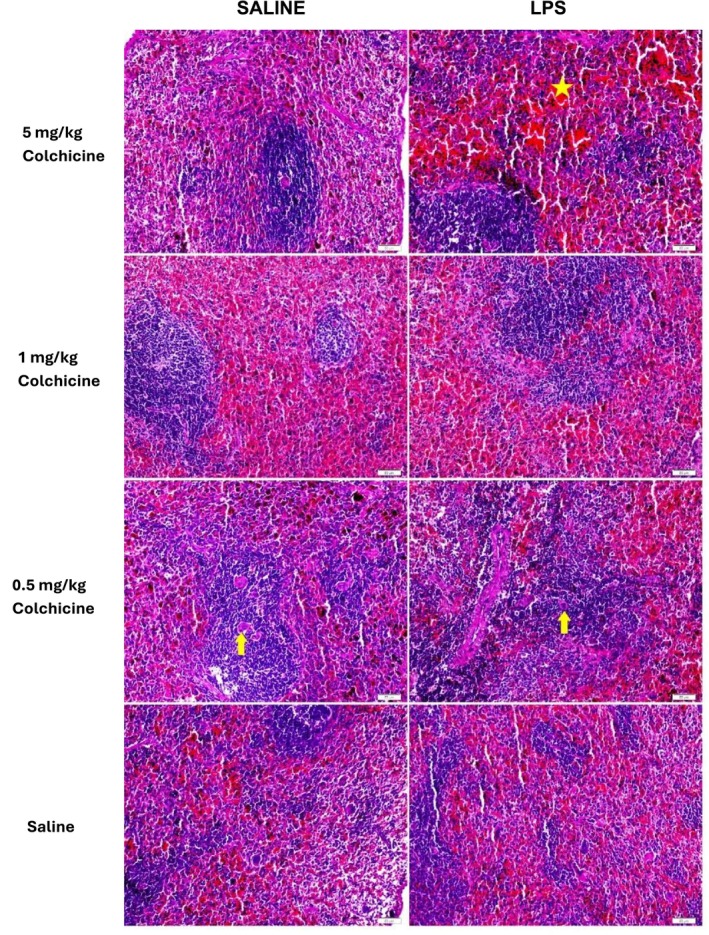
Effect of different colchicine doses on histopathological changes in the rat LPS induced endotoxemia model. Spleen sections from treatment groups and control experimental animals were compared using the H&E staining method (yellow arrow: lymphocyte, orange star: bleeding). Magnification 40×, bar = 20 μm.

The histopathological spleen damage score was significantly increased in the LPS group compared with the control group (Figures 4 and 5, *p* < 0.0001). Treatment with colchicine at a dose of 1 mg/kg significantly reduced the LPS‐induced splenic damage; however, the scores did not reach control levels. In contrast, colchicine at 0.5 mg/kg did not significantly affect the histopathological damage score, whereas the 5 mg/kg dose further exacerbated splenic injury compared with the LPS group (*p* < 0.05). According to the statistical analysis, no significant differences were observed in wet spleen weights among the groups.

## 4 Discussion

This study evaluated the dose dependent effects of the anti‐inflammatory colchicine in an LPS‐induced experimental endotoxemia model in rats. Intraperitoneal administration of 1 mg/kg LPS was selected because it reliably induces a rapid and reproducible systemic inflammatory response, characterized by cytokine release and hemodynamic alterations, while avoiding confounding effects of active infection [18, 19]. This model therefore represents non‐infectious endotoxemia rather than true clinical sepsis. Unlike previous studies that focused on isolated inflammatory or organ‐specific endpoints, the present work integrates systemic cytokine profiling, mesenteric perfusion assessment, and parallel histopathological evaluation of the liver and spleen within the same experimental framework, enabling a more comprehensive interpretation of dose dependent colchicine effects.

LPS administration is known to induce a rapid increase in pro‐inflammatory cytokines, with serum TNF‐α levels becoming readily detectable within approximately 3–4 h after challenge and remaining markedly elevated during the acute phase of endotoxemia [20, 21].

In the present study, colchicine was administered 1 h after LPS injection, and mesenteric arterial blood flow together with serum concentrations of IL‐1β, IL‐6, IL‐8, IL‐10, and IL‐17 were assessed at 4 h after LPS challenge. Importantly, mortality was observed in the LPS + 5 mg/kg colchicine–treated group, with two animals dying before completion of the experimental protocol, resulting in a reduced effective sample size. Although the precise cause of death could not be definitively determined, gross post‐mortem examination revealed markedly stiff and contracted cardiac tissue, consistent with a potential dose‐related cardiovascular toxic effect. It is plausible that the systemic inflammatory milieu induced by LPS enhances susceptibility to colchicine‐related adverse effects, as pre‐exposure to lipopolysaccharide has been shown to potentiate colchicine toxicity in rats. Endotoxemia is known to reduce hemodynamic reserve, alter vascular reactivity, and increase susceptibility to drug‐induced cardiovascular depression; therefore, doses that may be tolerated under non‐inflamed conditions can become toxic in the presence of systemic inflammation [22]. Accordingly, our findings indicate that although colchicine exerts anti‐inflammatory effects across a range of doses, its therapeutic window in experimental endotoxemia is narrow. The cardiovascular instability and mortality observed at the 5 mg/kg dose are consistent with previous reports describing increased colchicine toxicity at higher doses in rats [22, 23, 24]. Taken together, our results reinforce that colchicine exhibits an exceptionally narrow therapeutic window in acute systemic inflammation.

Although gross cardiac abnormalities were observed in animals that died following high‐dose colchicine, detailed histopathological or molecular analyses of cardiac tissue were not performed, which limits mechanistic interpretation of the observed cardiovascular toxicity and should be addressed in future studies.

The earliest hemodynamic alterations during systemic inflammation are frequently observed in the mesenteric circulation [4, 25]. During endotoxemia, reduced mesenteric blood flow primarily reflects microcirculatory dysfunction and maldistribution of perfusion rather than simple vasoconstriction. In the early phase, this reduction may initially represent a compensatory redistribution of blood toward vital organs; however, when sustained, mesenteric hypoperfusion can contribute to intestinal ischemia, disruption of gut barrier integrity, bacterial translocation, and subsequent amplification of systemic inflammatory responses [26, 27].

In this context, colchicine would not be expected to act as a direct vasodilator, but rather to improve mesenteric perfusion indirectly by attenuating inflammation‐driven endothelial dysfunction and microvascular obstruction. In our study, mesenteric blood flow was significantly increased only at the 5 mg/kg colchicine dose, whereas lower doses failed to restore mesenteric perfusion. This finding suggests that only higher colchicine exposure was sufficient to counteract the profound microcirculatory disturbances induced by LPS, whereas lower doses may have been inadequate to overcome endotoxemia‐associated perfusion deficits. In addition, microtubule disruption with colchicine has been shown to enhance β‐adrenoceptor–mediated vasorelaxation in isolated rat mesenteric arteries, with evidence for an increased β₂‐adrenoceptor contribution; thus, enhanced β₂‐mediated vasodilatory responsiveness may have indirectly contributed to the improved mesenteric blood flow observed at the highest dose [28, 29]. Notably, despite improving mesenteric perfusion, the 5 mg/kg colchicine dose was associated with increased mortality and tissue injury, indicating that restoration of regional blood flow alone is insufficient to confer overall protection and may occur in parallel with systemic toxicity at excessive exposure.

LPS‐induced endotoxemia leads to the release of TNF‐α and other pro‐inflammatory cytokines, which disrupt endothelial junctional integrity and increase capillary permeability, thereby contributing to endothelial injury and systemic inflammatory responses [30].

In addition, cytokines such as IL‐8 and IL‐17 are upregulated during LPS‐induced endotoxemia. IL‐8 plays a central role in neutrophil chemotaxis, whereas IL‐17 promotes the activation and recruitment of multiple immune cell populations, thereby amplifying innate, adaptive, and humoral immune responses [31, 32]. In the present study, the 1 mg/kg colchicine dose was the only regimen that effectively reduced serum concentrations of IL‐1β, TNF‐α, IL‐8, and IL‐17, indicating that this dose achieves an optimal balance between anti‐inflammatory efficacy and tolerability under endotoxemic conditions.

IL‐6 plays a complex and context‐dependent role in systemic inflammation [33]. Although elevated IL‐6 levels are frequently associated with disease severity and increased mortality in clinical sepsis cohorts [34], IL‐6 also exerts important regulatory and anti‐inflammatory effects by suppressing the release of pro‐inflammatory cytokines such as TNF‐α and IL‐1β and by promoting anti‐inflammatory pathways [35].

In our study, LPS administration significantly increased serum IL‐6 levels. While none of the colchicine doses fully normalized IL‐6 concentrations to control levels, colchicine at 0.5 and 1 mg/kg reduced IL‐6 levels compared with the LPS group. In contrast, IL‐6 concentrations were markedly elevated in the 5 mg/kg colchicine group, suggesting that excessive colchicine exposure may contribute to inflammatory dysregulation and may serve as an indicator of dose‐related toxicity.

IL‐10, a potent anti‐inflammatory cytokine, limits excessive inflammatory responses by suppressing the production of pro‐inflammatory mediators [36]. However, although IL‐10 is generally considered protective, elevated IL‐10 levels have also been associated with worse outcomes in severe systemic inflammation, reflecting immune dysregulation or immunoparalysis [37]. In the present study, colchicine treatment reduced IL‐10 concentrations, with the most pronounced effect observed at the 1 mg/kg dose. While the clinical implications of IL‐10 modulation remain complex, these findings may indicate a more balanced immune response at this dose.

Cytokine levels were also assessed in tissue homogenates obtained from the liver, spleen and heart; however, no statistically significant differences in tissue cytokine concentrations were detected between control and treatment groups. The absence of detectable tissue‐level changes may be attributable to both biological and technical factors. In experimental endotoxemia, circulating cytokines such as TNF‐α, IL‐6, IL‐1β and IL‐10 rise rapidly after LPS exposure, with serum concentrations typically peaking within 2–4 h [20]. In contrast, cytokine responses in solid organs are often earlier and more transient. Experimental studies in rats have demonstrated that tissue TNF‐α and IL‐1β levels—particularly in the liver—peak within 30–90 min following LPS administration and subsequently decline due to local receptor binding, intracellular degradation, and compartmentalization [21]. Accordingly, the 4‐h sampling time point used in the present study may have captured serum cytokines near maximal elevation, while tissue cytokine levels were already returning toward baseline.

In addition to these kinetic differences, technical limitations inherent to tissue cytokine quantification may have further contributed to the lack of detectable tissue level changes. Cytokine measurements in tissue homogenates are affected by dilution during homogenization, variable protease activity and protein‐rich matrices that can interfere with immunoassay performance. Recent methodological studies have demonstrated that tissue and tumor lysates generate substantial matrix effects, significantly reducing the sensitivity of ELISA and multiplex cytokine assays even under optimized experimental conditions [38]. Similarly, earlier work has shown that cytokine detection in tissue homogenates is markedly less sensitive than in serum due to dilutional loss and matrix interference [39]. Taken together, these kinetic and technical considerations suggest that modest tissue‐specific cytokine effects of colchicine may have been present but remained below the lower limit of quantification at this time point.

The liver and spleen represent key immunometabolic compartments during systemic inflammation, functioning both as major sources of inflammatory mediators and as primary targets of endotoxemia‐induced tissue injury. Given that the liver and spleen are major sources and targets of inflammatory mediators during endotoxemia, histopathological evaluation was performed to assess tissue‐level injury. LPS‐induced endotoxemia resulted in significant hepatic damage, characterized by hepatocellular injury, hemorrhage, inflammatory cell infiltration and increased histopathological damage scores. Among the colchicine‐treated groups, the lowest degree of hepatic injury was observed in the LPS + 1 mg/kg colchicine group, with damage scores not significantly different from those of the control group. These findings support a tissue‐protective effect of colchicine at this dose and are consistent with the corresponding improvements observed in serum cytokine profiles.

In parallel with hepatic injury, LPS‐induced endotoxemia also resulted in significant splenic damage, reflecting the central role of the spleen in systemic immune activation. Although none of the colchicine doses fully restored splenic histology to control levels, treatment with 1 mg/kg significantly attenuated splenic injury compared with the LPS group, whereas the 5 mg/kg dose further aggravated tissue damage. These findings suggest that, similar to the liver, the spleen is highly sensitive to both the protective and toxic effects of colchicine, reinforcing the concept of a narrow therapeutic window under endotoxemic conditions.

A notable limitation of the present study is the reduced statistical power in the 5 mg/kg colchicine+LPS group, as high‐dose toxicity led to 2 animal deaths and consequently a smaller effective sample size, which may have increased variability and limited the robustness of statistical comparisons for this dose. In addition, the LPS‐induced endotoxemia model reflects acute sterile inflammation and does not fully recapitulate the complexity of polymicrobial clinical sepsis, which should be considered when interpreting the translational relevance of our findings.

Furthermore, measurements were obtained at a single early time point (4 h) after LPS challenge, precluding assessment of delayed, progressive, or recovery phase effects of colchicine on inflammation, organ injury and hemodynamics. Finally, although three different colchicine doses were evaluated, the non‐linear dose–response relationship observed suggests that testing additional intermediate doses may further refine the definition of the therapeutic window and help delineate the boundary between efficacy and toxicity in this model. Future studies should also address the effects of repeated or chronic colchicine administration on efficacy, toxicity and translational relevance in systemic inflammatory conditions.

Collectively, our data indicate that colchicine exhibits a clear dose‐dependent dual effect in LPS‐induced endotoxemia, exerting anti‐inflammatory and tissue‐protective actions at lower doses while leading to cardiovascular instability and mortality at higher exposure. Although colchicine exerts anti‐inflammatory effects across a range of doses, its therapeutic window is narrow, with the 1 mg/kg dose providing the most consistent balance between efficacy and safety in this model. In contrast, the cardiovascular instability and mortality observed at the 5 mg/kg dose are consistent with experimental evidence demonstrating dose‐dependent colchicine toxicity in rats and further suggest that systemic inflammatory stress may lower the threshold for adverse cardiovascular effects [22, 23, 24].

From a translational perspective, the identification of a dose that simultaneously suppresses systemic inflammation and limits organ injury without overt toxicity underscores the importance of careful dose selection when repurposing colchicine for acute inflammatory conditions. Although direct extrapolation to humans is not warranted, these findings highlight that, if colchicine is considered in patients with sepsis or other severe systemic inflammatory conditions, careful dose selection, adjustment, and close monitoring may be critical to avoid toxicity and underscore the importance of defining an optimal therapeutic range when repurposing colchicine for acute inflammatory diseases.

## 5 Conclusion

Colchicine is a well‐established anti‐inflammatory drug; however, its clinical utility is limited by a narrow therapeutic window and dose‐related toxicity. Therefore, the present study aimed to define the optimal effective dose and to delineate the dose‐dependent balance between efficacy and toxicity of colchicine in a rat model of LPS‐induced experimental endotoxemia.

Wet liver and spleen weights, mesenteric artery blood flow, systemic and tissue cytokine profiles and histopathological damage scores were evaluated. Among the tested doses, colchicine at 1 mg/kg (i.p.) exerted the most consistent anti‐inflammatory effects, whereas the 5 mg/kg dose, despite partial hemodynamic improvement, failed to attenuate tissue injury and was associated with aggravated histopathological damage, indicating potential toxicity at higher exposure.

Collectively, these results demonstrate a dose dependent dual effect of colchicine in acute LPS‐induced endotoxemia and identify 1 mg/kg as the optimal anti‐inflammatory dose in this model. Importantly, the divergence between efficacy and toxicity underscores the critical role of dose selection. Although extrapolation to humans should be made with caution, these findings suggest that careful dose adjustment and close monitoring may be necessary if colchicine is considered in patients with sepsis or severe systemic inflammation. Further experimental and clinical studies are warranted to explore long‐term efficacy and safety.

## Author Contributions

**Meliksah Demir:** methodology, investigation, writing – original draft, data curation, software. **Ayse Yesim Gocmen:** methodology, investigation. **Esin Ozcelebi:** methodology, investigation, writing – original draft, data curation, formal analysis. **Zuleyha Doganyigit:** methodology, investigation, writing – original draft, visualization. **Aslı Okan Oflamaz:** methodology, investigation, writing – original draft, visualization. **Alper Bektas Iskit:** conceptualization, project administration, resources, writing – review and editing, funding acquisition, supervision, validation.

## Funding

This work was supported by Hacettepe Üniversitesi (Project ID: 19643).

## Conflicts of Interest

The authors declare no conflicts of interest.

## Data Availability

All data generated or analyzed during this study are included in this published article.
